# Breath holding duration as a measure of distress tolerance: examining its relation to measures of executive control

**DOI:** 10.3389/fpsyg.2013.00483

**Published:** 2013-07-29

**Authors:** Stefan Sütterlin, Mathias Schroijen, Elena Constantinou, Elyn Smets, Omer Van den Bergh, Ilse Van Diest

**Affiliations:** ^1^Research Unit INSIDE, University of LuxembourgLuxembourg; ^2^Research Group on Health Psychology, University of LeuvenLeuven, Belgium

**Keywords:** distress tolerance, breath holding, executive functions, self-regulation, emotion regulation

## Abstract

Recent research considers distress (in)tolerance as an essential component in the development of various forms of psychopathology. A behavioral task frequently used to assess distress tolerance is the breath holding task. Although breath holding time (BHT) has been associated with behavioral outcomes related to inhibitory control (e.g., smoking cessation), the relationship among breath holding and direct measures of executive control has not yet been thoroughly examined. The present study aims to assess (a) the BHT-task's test-retest reliability in a 1-year follow-up and (b) the relationship between a series of executive function tasks and breath holding duration. One hundred and thirteen students completed an initial BHT assessment, 58 of which also completed a series of executive function tasks [the Wisconsin Card Sorting Test (WCST), the Parametric Go/No-Go task and the N-back memory updating task]. A subsample of these students (*N* = 34) repeated the breath holding task in a second session 1 year later. Test-retest reliability of the BHT-task over a 1-year period was high (*r* = 0.67, *p* < 0.001), but none of the executive function tasks was significantly associated with BHT. The rather moderate levels of unpleasantness induced by breath holding in our sample may suggest that other processes (physiological, motivational) besides distress tolerance influence BHT. Overall, the current findings do not support the assumption of active inhibitory control in the BHT-task in a healthy sample. Our findings suggest that individual differences (e.g., in interoceptive or anxiety sensitivity) should be taken into account when examining the validity of BHT as a measure of distress tolerance.

## Introduction

The definition of distress tolerance as *persistence* to a stressor (Brown et al., 2002) or *withstanding* distress (Leyro et al., 2010; Zvolensky et al., 2010) implies an active overcoming of an unpleasant experience and as such it is considered to be different from (although overlapping with) emotion regulation (Leyro et al., 2010). Distress (in)tolerance has recently gained more attention as it is put forward as a crucial concept in both the etiology and course of various forms of psychopathology (e.g., anxiety, depression, substance or behavioral addictions and chronic pain; Zvolensky et al., 2010). The rapid increase of research in this area was followed by an increasingly critical appraisal of the boundaries of this concept and its relations to related research areas such as emotional and self-regulation (Leyro et al., 2010; Zvolensky et al., 2010). Recent publications critically discussed both the ambiguity of the definition of distress tolerance, as well as the conceptual validity of various assessment techniques such as self-report or experimental distress induction (Leyro et al., 2010; McHugh et al., 2010). Therefore, more research on the conceptual match between assessment methods and the construct of distress tolerance is needed.

The concept of distress tolerance is used in literature in order to refer to both a “perceived capacity to withstand negative emotional and/or other aversive states” (Zvolensky et al., 2010) and the “behavioral act of withstanding distressing internal states elicited by some type of stressor” (Zvolensky et al., 2010). It is thus commonly assessed via both self-report measures and behavioral tasks involving the experimental induction of distress. One of the most frequently used behavioral indices for distress tolerance is the breath holding task (e.g., Brown et al., 2002). In this task, participants are asked to hold their breath as long as possible (an experimenter records the actual breath-holding time, BHT) with precise instructions varying in literature (e.g., “hold your breath as long as possible,” “hold your breath until you feel the urge to breath”)^1^.

In their recent review, Leyro et al. (2010) pointed out numerous shortcomings in the currently common conceptualizations of distress tolerance and their particular operationalizations such as the breath holding task. The question of whether the ability to voluntarily hold one's breath measures rather trait-like global distress tolerance ability or a more context-specific ability related to anxiety interacting with bodily sensations remained so far unanswered. Previously reported associations with trait characteristics like trait anxiety, fear of suffocation (Eke and McNally, 1996; Eifert et al., 1999) and neuroticism (Johnson et al., 2011), within-session comparisons (Bernstein et al., 2008) and correlations (Johnson et al., 2011) of sequential BHT as well as a considerable contribution of physiological (co-) determinants to maximum breath holding time (BHT) (McKay et al., 2008) suggest a high trait component of this measure. Nevertheless, its test-retest reliability has not been previously examined.

Repeated assessments of BHT suggested high short-term stability (*r* = 0.82, Johnson et al., 2011; cf. Bernstein et al., 2008). However, these repeated measures were assessed within-session, leaving enough room for state-dependent (arousal, anxiety, mood, etc.) influences. In spite of the still scarce knowledge about the underlying mechanisms determining breath holding duration in healthy samples, the first aim of this study was to examine the reliability of the task. To achieve a more reliable measure of test-retest reliability, a long-term comparison was carried out, collecting BHTs under identical conditions in a 1-year follow-up. Findings on the reliability of the task are of high relevance for the supposed trait-like character of the capacity to hold one's breath and can provide useful insights in fields where the BHT has been used as a predictive variable for therapy outcomes or as a risk factor (e.g., relapse probabilities during post-treatment distress). Based on these previous findings, we hypothesized a high positive test-retest-correlation of BHTs. Confirmation of temporal stability of BHT measures would support the assumption of it as a personality trait (as opposed to be determined by motivational/attentional predictors).

As the definition of distress tolerance implies a process of actively overcoming an unpleasant experience and thus emotional and/or behavioral control, a task to measure distress tolerance could be expected to tap into self-regulatory resources. Self-regulatory functions required for the down-regulation of negative affect are known to involve active inhibitory processes (Ochsner et al., 2004; Mueller, 2011) involving prefrontally originating inhibitory projections to subcortical structures (Goldberg, 2001; Miller and Cohen, 2001; Banfield et al., 2004; Casey et al., 2008). Alpher and Blanton (1991) suggested that the function of a behavioral inhibition system (BIS; Gray, 1982) plays a crucial role when individuals face the inhibitory demands of the breath holding task. This suggestion is supported by studies linking the breath holding task to behavioral outcomes like smoking cessation (Hajek et al., 1987; Hajek, 1991; Zvolensky et al., 2001; Brown et al., 2002; MacPherson et al., 2008), relapse risk in pathologic gamblers (Daughters et al., 2005) and panic disorder (Asmundson and Stein, 1994). In the context of panic disorder, psychological factors such as anxiety sensitivity and fear of suffocation played an important role in panic patients' ability to hold their breath as long as possible (Eke and McNally, 1996; Roth et al., 1998; Eifert et al., 1999; Brandt et al., 2012).

Further evidence for a link among breath holding and the activation of BIS comes from studies using the breath holding task as a correlate of effortful behavioral self-regulation. Specifically, BHT was shown to be reduced after depletion of self-regulatory resources during unpleasant (distressing) sensations (Muraven et al., 1998; Vohs and Schmeichel, 2003), as predicted by the limited resource model of self-regulation (Vohs et al., 2009). More recent research, however, proposed alternative explanations for ego-depletion models involving motivational and attentional processes. These new accounts focus on the role of impulses rather than their regulation (Inzlicht and Schmeichel, 2012). Following this approach, ego-depletion can be explained without associations with behavioral measures of self-control such as the breath holding task and underline the need for a validation of the breath holding task as a reliable measure for effortful self-control in healthy samples.

If the breath holding task indeed involves an active and effortful withstanding to aversive stimuli, breath holding duration should be associated with performance on neurobehavioral tests loading on cognitive and/or behavioral inhibitory resources. However, there has so far been no thorough investigation of the relationship among breath holding and executive control or, more specifically, inhibitory function. Although neuro-imaging findings suggest overlapping neural areas during breath holding and motor response inhibition (McKay et al., 2008), the suggestion of an actual overlap among behavioral measures of both concepts has not been investigated. The BIS has previously been associated with activation in prefrontal inhibitory areas (Shackman et al., 2009), but in how far this correlation is related to BHT remains unclear. This is particularly striking given the high relevance of executive functions (in terms of prefrontal disinhibition) in psychopathology related to behavioral or emotional dysregulation (Mueller, 2011). Specifically, numerous studies have linked low performance in measures of executive functions with substance addiction, behavioral addiction, eating disorders and depression (Tekin and Cummings, 2002), which in turn have also been associated with decreased distress tolerance. Thus, a second aim of this study was to systematically investigate the relationship between breath holding duration (under the assumption of high temporal stability of the BHT) and a broad selection of classical executive function tasks tapping into trait-like capacities of inhibitory processes. Although inhibition has been shown to rely on common neural resources in the right inferior frontal cortex (for an overview see Aron, 2007; Dillon and Pizzagalli, 2007), it comprises various components working in concert whilst different assessment methods tap into different resources (Barkley, 1997; Casey et al., 1997). We therefore used a combination of measures of cognitive flexibility (Wisconsin Card Sorting Test, WCST), pre-potent motor response inhibition (Go/No-Go), and working memory updating (N-back), all of them resembling typical assessment methods measuring inhibitory processes involved in self-regulation (Konishi et al., 1998, 1999; Hansen et al., 2004; Aron, 2007; Dillon and Pizzagalli, 2007).

To sum up, prior research tends to interpret BHT as a correlate of effortful behavioral self-regulation, and parallels with the BIS concept are suggested. Furthermore, distress tolerance shows conceptual similarity with the active down-regulation of negative affect, involving inhibitory control. Therefore, we hypothesized that breath holding duration is (a) a temporally stable trait and (b) as such correlates positively with indices of executive control. Although BHT has not been validated in healthy samples with standard neuropsychological assessment tools, as those applied here, we expected to find positive associations similar to previous studies in which health-related behavior (e.g., smoking cessation) was predicted. At the same time, we expected breath holding duration to be inversely correlated with anxiety sensitivity as it has been previously reported (Johnson et al., 2011), while its relation to other relevant constructs like Negative Affectivity and habitual symptom reporting were assessed in an exploratory manner and therefore no specific hypotheses were formulated.

## Methods

### Sample

The sample consisted of first year psychology students who participated in a collective psychological testing as a part of their methodology course. Testing consisted of various separate sessions throughout the academic year including a session of group questionnaire completion, a session of group computer testing and one group assessment of BHT. A total of 113 students (95 women, 18 men, *M* = 19.41 years, *SD* = 0.85, age range = 18–25 years) completed the BHT session, 58 of which had also completed the entire series of executive function tasks during the computer testing. A subsample of these students (*N* = 34, 2 men, *M* = 20.2 years, *SD* = 0.49) repeated the breath holding task in a second group session (identical to the first one) 1 year later to examine the test-retest reliability of the BHT-measure.

### Tasks

#### Executive function tasks

Executive functioning was examined with three tasks assessing three components of executive functions i.e., inhibition of prepotent responses, cognitive flexibility and updating in working memory. While representing different subcomponents of executive functions, all three tasks have been shown to tap into common neural resources related to prefrontal inhibition and have been shown to be involved in cognitive or behavioral control required for emotion regulation processes (e.g., Aron, 2007; Dillon and Pizzagalli, 2007).

– *Response inhibition* was assessed with the Parametric Go/No-Go task (PGNG; Langenecker et al., 2007). The PGNG task is a computerized reaction time (RT) task consisting of three different levels of difficulty. For each level participants see a series of letters (black small case letters on a white background) presented one after the other at the center of the screen for 600 ms each (no inter-stimulus interval). At Level 1, participants are asked to press a specified button (“*n*”) as soon as possible every time one of three target letters (*x, y, z*) appears on screen (“GO” level). At Level 2 (“Go/No-Go level”), participants are asked to respond to two targets (*x* or *y*) every time they appear in alternate order, but inhibit their response when the current target is the same as the previous responded target (i.e., after responding to an “*x*,” you can respond to a “*y*” but not to another “*x*”). Level 3 follows the same rule but increases in difficulty since participants have now three targets (*x, y, z*) to respond (or inhibit their response) to. Levels 2 and 3 are considered to pose increasing inhibitory demands and accuracy at the No-Go trials of these two levels is considered to load on a common inhibitory control factor (Votruba and Langenecker, 2013). Thus, the mean percentage of correct responses at Level 2 and Level 3 is used in the study as an index of behavioral inhibition. RTs were omitted from analyses since group computer testing does not allow for accurate RT recording.
– *Cognitive flexibility* was measured by a computerized version of the WCST (Berg, 1948). During this task, participants are presented with a series of cards that can be matched based on three rules: the number, the shape or the color of the elements on the card. At each trial participants see a series of four cards at the top of the screen, and have to choose which of the four matches a fifth card (the response card), based on one of the three rules. The correct rule is not revealed to the participants; rather they are asked to figure it out based on the feedback they receive upon response and are told that the rule may change without notice. The rule actually changed every time participants completed ten consecutive correct trials. The task finished when participants completed six blocks (got each of the three rules twice) or after 128 trials. As indices of flexibility, the number of categories completed and the number of perseverative errors (i.e., errors due to a failure to switch from the previous rule) were computed.
– *Updating* in working memory was assessed with the N-back task. The N-back task is a continuous performance task of various levels of difficulty that requires updating of working memory (Braver et al., 1997). During this task participants view series of letters (black capital letters on a white background) presented one after the other for 500 ms (1800 ms inter-stimulus interval) and are asked to respond to a target. Two levels, 0-back and 3-back, were used for this study. At 0-back, participants are asked to merely respond as fast as possible every time they see a target letter (“*x*”). At 3-back participants have to respond whenever the current letter is the same as the one presented three trials back. Participants completed two blocks for each level consisting of 30 trials each (the first three trials of each block were excluded from analyses), ten of which were target trials (to be responded). Total accuracy (average percentage of correct responses in target and non-target trials) was calculated for 0-back and 3-back separately.

#### Breath holding task

Breath holding was assessed in a group manner, requiring modifications to the standard breath holding procedure. Participants were seated in a large auditorium and were instructed to hold their breath after complete expiration (they were specifically asked not to alter their breathing prior to breath holding and thus asked to hold their breath after a full, normal, expiration). While holding their breath, they were asked to keep their eyes closed. In order to maximize experienced distress the participants were instructed to hold their breath “as long as you can, even if you feel the urge to breathe again.” To calculate BHTs, participants were asked to record the starting time from a timer projected in the room as soon as they held their breath (and just prior to closing their eyes) and the ending time as soon as they breathed in again and opened their eyes. The difference score between the ending time and the starting time was used in analyses.

### Self-report measures

#### Anxiety sensitivity

Anxiety sensitivity, i.e., the fear of anxiety-related bodily sensations due to beliefs about possible physical, social and mental consequences, was assessed by the Anxiety Sensitivity Index- 3 (ASI-3; Taylor et al., 2007). ASI-3 is an 18-item questionnaire, which assesses the degree people endorse statements about their reaction toward bodily arousal on a 5-point Likert scale (0 = very little, 4 = very much), e.g., “It scares me when my heart beats rapidly.” Three sub-scales of anxiety sensitivity are included in this questionnaire, namely physical concerns, cognitive concerns and social concerns. Total anxiety sensitivity scores (ranging from 0 to 72) were calculated.

#### Habitual symptom reporting

Participants' tendency to report symptoms in everyday life was assessed with a 39-item questionnaire, the Checklist for Symptoms in Daily Life (CSDL, Wientjes and Grossman, 1994), which assesses on a 5-point Likert Scale (1 = never, 5 = very often) the extent to which people experienced a series of physical symptoms over the past year. Symptoms of various modalities as well as dummy symptoms are included and the total score of all items (ranging from 39 to 195) was used in analyses. The measure has been found to have acceptable reliability exceeding the criterion of >0.70 (Wientjes and Grossman, 1994).

#### Trait negative/positive affectivity

Participants' tendency to experience positive or negative affect was assessed via the Positive and Negative Affect Schedule (PANAS, Watson et al., 1988; Dutch version validated by Engelen et al., 2006). The PANAS includes ten positive adjectives (e.g., strong) and ten negative ones (e.g., afraid) and participants must indicate the extent to which each adjective describes how they feel in general on a 5-point Likert Scale (1 = very slightly, 5 = very much). Total scores for the two subscales, Positive and Negative Affectivity were computed.

#### Breath holding task-experience questionnaire

Following the breath holding task, a purpose-made questionnaire was administered to the participants in order to check for possible covariates linked to the BHT. The questionnaire included questions on demographic information (sex, age, exercise/sports, smoking, medication/health, BMI, menstrual cycle for women), as well as questions regarding participants' experience and compliance with the breath holding task in a retrospective way. Specifically, participants had to rate on a 9-point Likert scale (a) how pleasant/unpleasant they felt during the task (1 = very unpleasant, 9 = very pleasant), (b) to what degree they followed the instructions precisely (1 = not at all, 9 = perfectly), (c) to what degree they tried to hold their breath as much as possible (1 = not at all, 9 = very much) and (d) whether they could hold their breath even longer (1 = definitely not, 9 = certainly). Finally, to control for individual differences in coping behaviors during the task participants had to report their thoughts and possible strategies used during breath holding.

### Procedure

Students completed three different sessions of collective testing throughout their first year of studies. The first session was the questionnaire assessment, conducted in a large auditorium where students were seated and completed a series of questionnaires in paper-and-pencil format for about 60 min (those presented here are only a part of the assessment). The second session was the executive function task session conducted in the computer labs of the university. Students were seated in front of desktop computers (in groups of 20), then were given instructions for the three tasks and started completing them at individual pace one after the other. To control for fatigue, the three tasks were counterbalanced, i.e., six orders were created so that each task was presented twice in each position. E-prime 1.0 (Schneider et al., 2002) was used for the presentation of the PGNG and the N-back tasks, while WCST was completed on-line via the Inquisit 3 Web software (Seattle, WA: Millisecond Software). Finally the breath holding task was conducted in a separate session about 6 months after the other two, in a large auditorium allowing for students to sit as far as possible from each other. Students were given the BHT-experience questionnaire in advance and were asked to complete the breath holding task whenever ready as soon as a projected timer started. One year later the same participants were re-administered the breath holding task in the same auditorium with the exact same procedure. Students received either course credit or entered a lottery for small prices.

### Statistical analysis

Pearson's *r* bivariate correlations and intraclass correlations (confidence interval 95%, two-way random) were calculated to assess test-retest stability. Pearson's *r* bivariate correlations were conducted to examine the relationships among BHT and psychological variables, while independent sample *t*-tests were used to investigate possible differences in BHT due to various physiological or demographic factors. Probability level was set to 0.05. All analyses were conducted with STATISTICA 10.0 software (StatSoft/Germany).

## Results

### Breath holding time (BHT)

**Figure 1:**
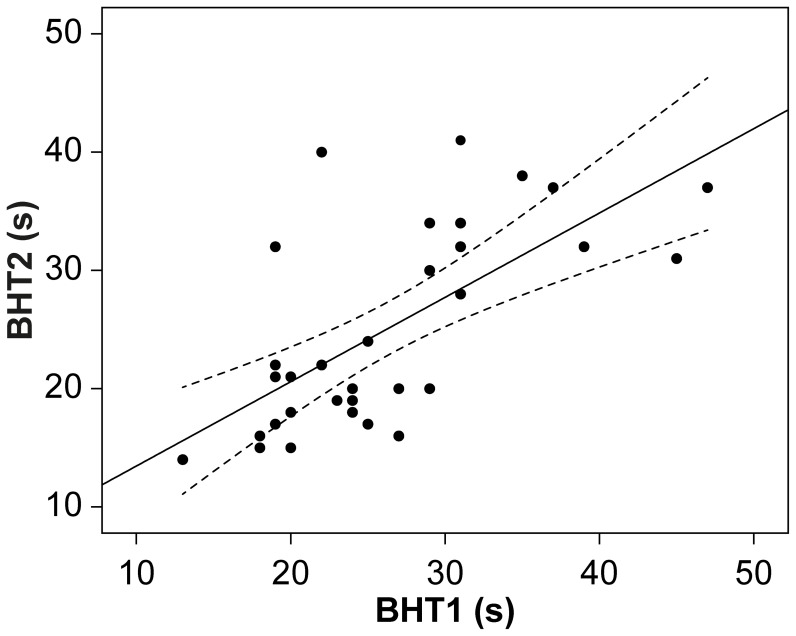
**Scatter-plot with breath holding times of both measurements (*N* = 33).** Note: Curves represent confidence intervals to the mean.

BHTs above 60 s were identified as outliers, resulting in the exclusion of three of 113 participants for the first breath holding test (BHT1) and one of 34 for the retest session (BHT2). Mean BHTs (presented in Table 1) did not differ significantly between test and retest [*t*_(32)_ = 1.07, *p* = 0.29] and are comparable (although slightly lower) with those reported by other studies using the standard BHT procedure with student samples (Eke and McNally, 1996; Eifert et al., 1999)^2^. Test-retest reliability over a 1-year period is high, as indicated by a positive Pearson correlation between BHT1 and BHT2 [*r*_(31)_ = 0.67, *p* < 0.001], see Figure 1. Intra-class correlation coefficients were high in consistency [average measures ICC(C.2) = 0.799; single measures ICC(C.1) = 0.665] and in absolute agreement [average measures, ICC(A.2) = 0.798; single measures ICC(A.1 = 0.664)].

**Table 1:** **Descriptive statistics for participants' performance and self-report**.

**Variables**	***N***	**Mean**	**Minimum**	**Maximum**	***SD***
**BREATH HOLDING**					
BHT1	110	26.75	12	54	8.85
BHT2	33	25.15	14	41	8.38
**Self-report**					
Positive affect	54	32.67	19	44	4.91
Negative affect	54	25.09	14	40	5.94
Habitual symptom reporting	54	93.18	59	139	18.45
Anxiety sensitivity	97	18.66	0	66	10.97
**Executive function tasks**					
Go/NoGo—L2 and L3 accuracy	56	60.77	16.90	92.85	16.10
WCST—number of categories	58	5.27	1.00	6.00	1.28
WCST—perseverative errors	57	22.75	5.00	65.00	14.97
0-back—total accuracy	55	95.18	76.67	100.00	4.90
3-back—total accuracy	55	76.57	36.67	100.00	12.28

### Potential correlates of BHT

To assess the relationship of breath holding ability with physiological and psychological variables, Pearson's product-moment correlations were calculated using the data from the first breath holding session (BHT1) only. BHT1 did not correlate with body mass index (BMI), *r*_(110)_ = 0.06, *p* = 0.50, or age, *r*_(110)_ = −0.06, *p* = 0.50. An independent samples *t*-test comparing smokers and non-smokers indicated no effect of smoking, *t*_(107)_ = 0.08, *p* = 0.93. Among smokers, smoking frequency (daily/weekly/seldom) did not relate to BHT either, *rho*(15) = −0.18, *p* = 0.53^3^. However, the small number of smokers does not allow firm conclusions. Participants that exercise regularly tended to have longer BHT than those who don't, as indicated by *t*-tests, *t*_(110)_ = 1.69, *p* = 0.09. *T*-test analyses indicated no significant sex differences in BHT [Men: *M* = 29.4, *SD* = 10.8; Women: *M* = 26.3, *SD* = 8.5; *t*_(108)_ = 1.28, *p* = 0.20, *d* = 0.32]. Executive functioning tasks did not correlate with BHTs (see Table 2).

**Table 2:** **Pearson's product-moment correlations among BHT and executive function tasks**.

	**2**	**3**	**4**	**5**	**6**
1. BHT1	−0.04 (*N* = 54)	0.19 (*N* = 56)	−0.03 (*N* = 55)	0.14 (*N* = 53)	−0.01 (*N* = 53)
2. PGNG—L2 and L3 accuracy		−0.04 (*N* = 56)	−0.01 (*N* = 55)	0.20 (*N* = 53)	0.42^*^ (*N* = 53)
3. WCST—number of categories			−0.65^**^ (*N* = 57)	0.18 (*N* = 55)	−0.09 (*N* = 55)
4. WCST—perseverative errors				0.01 (*N* = 54)	0.13 (*N* = 54)
5. 0-back—total accuracy					0.43^*^ (*N* = 55)
6. 3-back—total accuracy					

* p < 0.01;

** p < 0.001.

None of the questionnaires assessing trait characteristics correlated with BHT. As expected, there was a trend toward a negative association between anxiety sensitivity and BHT1, *r*_(94)_ = −0.18, *p* = 0.075. Even though the correlation is small, it may indicate that anxiety sensitivity influences the relation between BHT and executive functioning. For further exploratory investigation, we split the sample into participants scoring high and low on anxiety sensitivity (median split: 17) and re-examined the correlation between BHT and WCST-number of categories (the index with the highest correlation). For the group with high anxiety sensitivity, a tendency toward a positive correlation was found [*r*_(26)_ = 0.35, *p* = 0.08, two-tailed], whereas no tendency was observed for the low anxiety sensitivity group [*r*_(26)_ = 0.12, *p* = 0.56, two-tailed]. The difference between both coefficients was not significant though (*Z* = 0.83, *p* = 0.20).

To examine their predictive value in conjunction, WCST-number of categories and total ASI scores were centered and entered as predictors in a multiple regression, as well as their interaction, with BHT as the dependent measure. The overall model was not significant [*R*^2^_*adj*_ = 0.005, *F*_(48)_ = 1.09, *p* = 0.36] and none of the predictors significantly explained BHTs (WCST-number of categories: beta = 0.19, *t* = 1.32, *p* = 0.19; total ASI: beta = −0.14, *t* = −0.80, *p* = 0.43; WCST-number of categories *x* total ASI: beta = 0.001, *t* = 0.001, *p* = 0.99).

Regarding participants' experience of and reaction toward the breath holding task, perceived unpleasantness during the breath holding task (*M* = 4.7, *SD* = 1.5 on a 1–9 scale) did not correlate with BHTs. Neither did the effort indices, nor did the compliance to given instructions correlate with BHT.

### Exploratory analyses

Twenty-five participants (22.9%) reported to have used a strategy for holding their breath during BHT1. Overall, these strategies fall within four categories: physical actions, relaxation, distraction and mindful focus on breath holding sensations. Physical actions include keeping the nose closed, holding the hands still, closing the eyes and swallowing. Relaxation inducing strategies reported were behaviors like “remaining calm” or “focusing on silence” while the distraction category contains actions to deliberately think of something else or to deviate the attention away from breathing. The category mindful focus on breathing sensations consists of strategies like a direct focus on holding their breath and thoughts about the accompanying sensations. Strategies regarding physical actions and distraction were the ones most used (respectively, 48 and 28% of reported strategies). *T*-tests indicated that participants using a strategy during the breath holding task exhibited significantly longer BHTs (*M* = 31.2, *SD* = 10.2) than those who did not (*M* = 25.4, *SD* = 8.1), *t*_(107)_ = 2.96, *p* < 0.005.

## Discussion

BHT has been used as a measure of distress tolerance and self-regulation in healthy and clinical samples. However, correlates and determinants of BHT in healthy samples are largely unknown, limiting the construct validity and specificity of this measure. In healthy samples, BHT was interpreted as a measure of distress tolerance (Leyro et al., 2010; Zvolensky et al., 2010; Brandt et al., 2012) and self-regulatory strength (Vohs and Schmeichel, 2003). To further investigate BHT as an assessment instrument for distress tolerance, this study focused on long-term test-retest reliability and the relationship between BHT and executive control in a healthy sample.

In this first long-term follow-up, breath holding duration showed to be a reliable measure over time and its high test-retest reliability suggests the determination of BHT by relatively stable trait characteristics comparable to common personality inventories with 1-year follow-up retests (Groth-Marnat and Mullard, 2010) or other peripheral physiological trait measures such as heart rate variability (Bertsch et al., 2012).

Results of the current study, however, seem to challenge the suggestion of Alpher and Blanton (1991) of an involvement of inhibitory systems in voluntary breath holding, as well as findings from more recent imaging studies showing activations in inhibition-related neural structures during breath holding (McKay et al., 2008). However, Alpher and Blanton (1991) also acknowledged an important role for cognitive, affective, and motivational factors triggering behavioral inhibition. It could be argued that the BIS (a system designed to promote the avoidance of punishment) during a breath-holding task is only activated in the context of considerable concomitant negative emotional distress. For example in the McKay et al. (2008) study, inhibition-related networks were activated during a fixed time window of 15 s of breath holding with additional CO_2_-enriched air application, which constitutes an atypical breath holding paradigm. Its fixed nature and the additional application of CO_2_ might have induced a higher level of distress compared to the more typical participant-controlled “as long as you can” instructions without additional CO_2_ induction. The exploratory statistical analyses indicating a role of worry in response to symptoms of arousal (measured by the anxiety sensitivity scale) seem to support this suggestion. It could be speculated that a sub-sample of persons scoring high in anxiety sensitivity would show a different result compared to our sample drawn from a general student population in which one can only expect a small proportion of highly anxious individuals.

According to our self-report data, experienced unpleasantness during a breath holding task as typically applied in research on distress tolerance and self-regulation is rather moderate in a healthy student sample. It could be hypothesized that executive control resources were not sufficiently activated, as these were not required due to a low level of perceived distress. The standard BHT procedure may be experienced as under full personal control which makes it difficult for strong stress responses to emerge in a highly functioning sample. It therefore seems that breath holding in a healthy student sample may not tap into inhibitory processes but rather be an index of other processes besides distress tolerance, such as physiological and motivational processes. The weak negative correlations between anxiety sensitivity and BHT may provide indications for the effect of such motivational processes although current findings do not allow the examination of such a claim due to the focus on a normal student population with a low prevalence of emotion-regulatory deficits.

Although the involvement of inhibitory processes in distress tolerance has been a central hypothesis in regards to individual differences in breath holding, alternative explanations remain possible. Hence, further research is required on the alternative determinants of breath holding. This includes research on the influence of inter-individual differences in viscero-sensation on breath holding duration in healthy samples as well as on other physiological mechanisms beyond motivation and emotion (McKay et al., 2008). Individuals with higher interoceptive sensitivity might feel more distressed during a breath holding task than others. The weak and statistically not significant negative association (*r* = −0.18) of BHT with anxiety sensitivity in this healthy sample is very similar to earlier findings (*r* = −0.13; Johnson et al., 2011). However, our sample consisted of healthy students, who scored overall quite low in anxiety sensitivity (comparably to other student samples, Taylor et al., 2007) and much lower than what has been reported in various clinical samples (Kemper et al., 2012). Exploratory analyses suggest that in a high anxiety sensitivity sub-group, BHT tended to correlate positively with cognitive flexibility (WCST). Even though the sub-group was rather small to provide conclusive findings, it may suggest that the relative contributions of psychological and physiological determinants of breath holding may be different in clinical and healthy samples and therefore the influence of such factors can be more prominent in clinical samples or selected non-clinical samples with relatively higher interoceptive sensitivity. The usefulness of BHT as a measure of executive functions and self-regulatory capacity cannot be completely disqualified on the basis of current data. Rather, current findings suggest that individual differences (like anxiety sensitivity) could moderate this relationship and future research taking these variables into account is necessary to confirm a possible link between BHT and executive functions.

Furthermore, the typical breath holding task (used in this study) did not induce a sufficient amount of unpleasantness (distress) in our healthy sample. Thus, motivational and self-regulatory resources were not required to an extent that produces sufficient inter-individual variance to account for differences in BHT. Future research assessing BHT as a measure of distress tolerance should consider increasing the threat value of the task itself by reducing the perceived control of the participant. One such example could be a combination of breath holding along with a mechanically induced full obstruction of breathing (occlusion), which is individually tailored based on previous BHT assessment. In such a paradigm (Pappens et al., 2012) participants would need to overcome a period of occlusion which is out of their own control and beyond their perceived level of tolerance. Such a design would induce an experimental modulation of the degree of experienced aversiveness and thus allow for a systematic investigation of the role of anxiety sensitivity.

Furthermore, the high test-retest reliability over a very long time-period and the absence of correlations with measures of executive functions or other trait characteristics leave the possibility of a strong contribution of purely physiological determinants (as opposed to psychophysiological or psychological variables). However, these were not investigated in the present study, nor were these factors typically discussed or investigated in studies on distress tolerance.

### Limitations

Firstly, the present study initially assumed a relationship between measures of executive functions and BHT. A wide range of executive function tasks loading on various aspects of inhibitory control (motor response inhibition, working memory updating, and cognitive flexibility) was applied. However, it is important to note that all tasks used neutral stimuli with low emotional significance for the participants, while during BHT the regulation of a rather unpleasant experience is required. The relationship of BHT with the emotional equivalents of these executive function tasks could differ from the one documented in this study, since behavioral control has previously been shown to interfere with simultaneous emotional cues (Herbert and Sütterlin, 2011). Furthermore, as the assessment of executive functions took place 6 months prior to the BHT, changes in these traits may have occurred and may have masked possible effects. Additionally, it was beyond the scope of this study to assess alternative explanations and other aspects of BHT. Previous research suggested an influence of individual differences in interoceptive sensitivity on emotional control also in healthy samples (Sütterlin et al., 2013). We did not investigate this relationship in the present study. Second, context-specific influences (group setting, effect of different verbal instructions) that might play relevant roles in healthy samples were not considered. The perception of dyspnea, fear of suffocation or negative body-related perceptions in general might be of increased salience in particular samples such as panic patients, but they should also be examined when the breath holding task is applied in healthy samples. In this first attempt to provide a detailed evaluation of this assessment technique from the perspective of executive control, we restricted our study on a healthy sample. Low effect sizes in this healthy sample and the group testing setting with potentially less controllable environmental influences might have contributed to our null findings. More subtle methods of self-regulatory assessment using endophenotypic markers could provide better insights in future studies (Sütterlin et al., 2010).

## Conclusions

The breath holding task is considered to be a behavioral indicator of distress tolerance and self-regulatory capacity. Until present, the concept of distress tolerance has not been clearly defined. In healthy samples, the breath holding task does not seem to share variance with other classical measures of self-regulation. Exploratory analyses indicates a possible role of anxiety sensitivity as a moderator of such a relationship. This would be in line with previous research in vulnerable and (sub)clinical samples. The BHT's stability over a 1-year period suggests that other trait-like characteristics may also be involved. Future research may want to investigate whether other inter-individual differences, e.g., interoceptive sensitivity or other physiological indices of self-regulatory ability, e.g., heart rate variability (Thayer and Friedman, 2002; Appelhans and Luecken, 2006), are associated with BHT.

### Conflict of interest statement

The authors declare that the research was conducted in the absence of any commercial or financial relationships that could be construed as a potential conflict of interest.

## Footnotes

^1^Numerous studies assess BHT after normal expiration, operationalizing distress tolerance based on the functional residual capacity estimate (the volume of air present in the lungs at the end of passive expiration; cf. Alpher and Blanton, 1991). Alternatively, participants are asked to hold their breath after a normal inspiration (cf. Hajek et al., 1987).

^2^The higher BHTs reported by Eke and McNally (1996; *M* = 30.2, *SD* = 10.1) and Eifert et al. (1999; *M* = 32.9, *SD* = 11.5) could be due to their procedure (recording BHT twice and selecting maximum scores) and/or the higher number of males in their samples.

^3^Spearman's rho was used since smoking frequency was assessed as an ordinal variable.
